# Physicochemical, Rheological, and Morphological Characteristics of Products from Traditional and Extrusion Nixtamalization Processes and Their Relation to Starch

**DOI:** 10.1155/2020/5927670

**Published:** 2020-01-29

**Authors:** Carlos Martín Enríquez-Castro, Patricia Isabel Torres-Chávez, Benjamín Ramírez-Wong, Armando Quintero-Ramos, Ana Irene Ledesma-Osuna, Jaime López-Cervantes, Jesús Enrique Gerardo-Rodríguez

**Affiliations:** ^1^Departamento de Investigación y Posgrado en Alimentos, Universidad de Sonora, Rosales y Blvd. Luis Encinas s/n, Centro 83000, Hermosillo, Sonora, Mexico; ^2^Universidad Autónoma de Chihuahua, Avenida Universidad y Pascual Orozco s/n, Universidad, 31110 Chihuahua, Mexico; ^3^Instituto Tecnológico de Sonora, 5 de Febrero 818 Sur, 85000 Ciudad Obregón, Sonora, Mexico

## Abstract

The aim of this study was to compare the physicochemical, rheological, and morphological characteristics of corn, nixtamalized flour, masa, and tortillas from the traditional nixtamalization process (TNP) and the extrusion nixtamalization process (ENP) and their relationship with starch. The traditional and extrusion processes were carried out using the same variety of corn. From both processes, samples of ground corn, nixtamalized flour, masa, and tortillas were obtained. The extrusion process produced corn flour with particle sizes smaller (particle size index, PSI = 51) than that of flour produced by the traditional nixtamalization process (PSI = 44). Masa from the TNP showed higher modulus of elasticity (*G*′) and viscosity (*G*^″^) values than that off masa from the ENP. Furthermore, in a temperature sweep test, masa from the TNP showed a peak in *G*′ and *G*^″^, while the masa from the ENP did not display these peaks. The ENP-produced tortillas had higher resistant starch contents and comparable firmness and rollability to those from the TNP but lower quality parameter values. A comparison of the products' physicochemical properties obtained by the two processes shows the importance of controlling the damage to starch during the milling and extrusion processes to obtain tortillas of better quality. For the first time, we propose the measurement of the viscoelastic parameters *G*′ and *G*^″^ in temperature sweep mode to monitor changes in the degree of starch damage.

## 1. Introduction

The processing of maize through nixtamalization has allowed the development of traditional products, such as tortillas and other innovators with high consumer acceptance [1]. Currently, through this process, the maize is cooked in a solution of Ca(OH)_2_, then soaked, washed, and ground, and thus to obtain flour, masa (dough), tortillas, and other products are widely acceptable.

During traditional nixtamalization structural changes in the grain occur, resulting in rheological, functional, and textural properties that determine the acceptability of the final product. Among the most important structural changes is the gelatinization of the starch that is affected by factors such as temperature-cooking times and wet milling operations [2, 3]. In the traditional nixtamalization process (TNP), wet milling is used to separate the starch granules with excess of water, which reduces the damaged starch content [4–7]. Both factors, moisture content and particle size, make gradual starch gelatinization possible, improving the viscoelastic behavior of the obtained masa, as well as the flexibility, rollability, firmness, structural uniformity, color, and shelf life of the tortillas; the sensory attributes are appreciated by the consumer. The lack of control of these factors (cooking conditions, grain moisture content, grinding particle size) in TNP results in a variability in the quality of nixtamalized products. Despite the benefits of traditional nixtamalization, this process involves high energy and water consumption, and leads to environmental pollution [8]. So alternative methods such as extrusion-cooking have been studied to obtain flour, masa, and tortillas [9–14].

Some studies of extrusion nixtamalization process (ENP) for obtaining nixtamalized corn flours, have evaluated the impact of the process conditions (temperature, moisture content, and calcium hydroxide, and enzymes in the feeding, screw speed, among others) in the physical and chemical properties of flour, masa, and tortilla, with good approaches to the traditional product [6, 7, 10–18]. However, adapting the ENP to obtain different quality nixtamalized products leads to technological limitations, since unlike wet milling, dry milling conditions used in ENP such as low water content and a reduced amount of lime, affect directly the behavior of corn starch, due to exhaustive mechanical force applied several times caused higher damaged starch contents [5–7, 19] and increase the content of starch damaged (SD).

Additionally, during extrusion-cooking an important damage occurs in starch granules, causing a certain dextrinization degree, depending on the ENP conditions [10, 16, 20]. So, viscoelastic properties are lower than those observed in TNP [13], affecting the quality of masa and tortillas from ENP. Despite these disadvantages of extruded nixtamalized flours, tortillas obtained with extruded nixtamalized corn flour have been claimed to be comparable in quality to those made using the TNP, though textural, and shelf life problems still exist. Thus, improvements have been focused on decreasing the firmness and increasing the rollability of tortillas [11, 14, 21–23].

These structural properties in tortillas are related to the changes that occur in starch, as described above. To determine which operations are critical in the ENP compared to TNP, it is essential to evaluate the changes that occur in starch and their impact on the quality of flour, masa, and tortilla. This allowed us to identify the extrusion conditions necessary to reduce the starch damage to a comparable level to that of the TNP.

The aim of this study was to evaluate and compare the physicochemical, rheological, and morphological characteristics of products from the traditional and extrusion nixtamalization processes and their relationship to changes in corn starch granules to gain knowledge to improve tortillas obtained with extruded flour.

## 2. Materials and Methods

### 2.1. Raw Material

White corn (*Zea mays L*.) H-430 and H-431 commercial hybrids were selected. This grain is resistant to high temperatures (up to 40°C) in the northwestern zone of México. It was purchased from a local market in Hermosillo, Sonora, México. Corn grain was cleaned using a vibratory cleaner (Clipper, Model V230, Clipper Products, Bluffton, IN, USA). The chemical composition of corn grain was determined following the AACC [24] International Approved Methods are as follows: 11.2% moisture content (44–15.02), 71.7% total starch content (dry basis), (76–13.01); 4.88% ether extract (dry basis), (30–20.01); 1.14% ash content (dry basis), (08–01.01); pH = 6.5 (02–52.01), and 9.17% (dry basis) protein content using the AOAC 990.03 method [25].

### 2.2. Extrusion Nixtamalization Process (ENP)

#### 2.2.1. Grinding

Three 2-kg samples of corn were ground individually with different meshes (0.5 mm, 0.8 mm, or 1 mm) in a hammer mill (Model FT2, Armfield Limited, Bridge House, West Street Ringwood, England). Grinding operation was according to procedure used by Escalante-Aburto et al. [26], and Platt-Lucero et al. [13]. In the same way, Tran et al. [27], relates the particle size index with the effect of starch damage during milling. A composed blend of ground corn was prepared by mixing 0.45 g/g of ground corn with 0.5 mm mesh, 0.40 g/g with 0.8 mm mesh, and 0.15 g/g with 1.0 mm mesh. This blend allowed an appropriate change (damage) in the starch granules to obtain a specific particle size for corn flour subjected to the extrusion process, and this change was made possible by manipulating the process factors in the extrusion experiment, including the type of mesh used to grind the grain, the moisture content of the feed material, the compression ratio, and screw speed.

#### 2.2.2. Conditioning

The extrusion process starts by grinding whole corn grain and conditioning with lime and water before entering the extruder. This step helps the water and lime diffuse into the internal structures of the corn kernel [11]. The blend of ground corn was supplemented with 0.3 g lime/100 g flour (Nixtacal Calhidra de Sonora; Hermosillo, México) and distilled water, and adjusted to 25 g water/100 g flour. This was mixed for 3 min in a horizontal mixer (Hobart model AS200; Troy, OH) and then stored in a refrigerator for 12 h at 5°C.

#### 2.2.3. Extrusion

A single screw laboratory extruder (Model E 19/25 D, OHG Duisburg, Germany) was used to obtain extruded nixtamalized corn flour (ENCF). The extrusion experiment was performed according to the procedure reported by Platt-Lucero et al. [13], but with different conditions. A 19 mm screw diameter was used, with a length-to-diameter ratio of 20 : 1, and a nominal compression ratio of 1 : 1, and a 3.0 mm die opening with four zone heating cooling (1300 W each). The feed speed of the conditioned sample was 45 rpm. The screw speed was 145 rpm, with an average residence time of 13 min, and an operating pressure of 5.68 atm (83.6 psi). The extrusion temperatures were 60°C, 70°C, 80°C, and 90°C in the first, second, third, and fourth heating/cooling zones, respectively. The extrudates were collected in aluminum trays for later drying.

#### 2.2.4. Drying and Grinding

The extrudates were dried at 60°C for 1 h using a custom-made constructed tunnel dryer (no brand) and according to the procedure reported by Platt-Lucero et al. [13]. The main objective of using the drying process was to reduce the moisture content in the extrudates coming out from the extruder. This moisture value lowered from 18% to 11%. Next, the dried extrudates were hammer-milled, first using a 0.8 mm mesh and then ground again by the mill with a 0.5 mm mesh to obtain the extruded nixtamalized corn flour.

#### 2.2.5. Tortilla Preparation

Tortilla were made in different ways depending on the nixtamalization process. In both processes of nixtamalization, the tortillas were prepared in a commercial plant (Tortillería Pimentel, Hermosillo Sonora, México).

For the ENP, once the extruded nixtamalized flour (ENCF) was obtained, the tortillas were prepared according to the procedure reported by Platt-Lucero et al. [13]. A sample of 2.5 kg of ENCF was mixed with water (2500 mL) in a horizontal mixer (Manufacturas Lenin Model 25, San Luis Potosi, Mexico) for 3 min to form 5.0 kg of corn masa as reported by Chaidez-Laguna et al. [12]. The amount of masa obtained in that mixer was the minimum that could be made (according to the operator) and the minimum amount to run and be processed in the tortilla machine roller. Masa was wrapped in a plastic bag and allowed to rest for 20 min before processing. Masa disks (25 g) were formed in a tortilla-forming machine (Lenin Manufactures, Model MLR 30, San Luis Potosí, Mexico) and baked for 56 s in a three step oven at temperatures of 221°C ± 10°C; 248°C ± 10°C, and 280°C ± 10°C. Corn tortillas were cooled and transported to the laboratory, where they were stored at room temperature (25°C) for further analysis.

### 2.3. Traditional Nixtamalization Process (TNP)

#### 2.3.1. Cooking and Steeping

Corn kernel samples (3 kg) were cooked with water (1 : 3) and 1 g lime/100 g, the grain was boiling (96°C) for 20 min according to the procedure reported by Ramírez-Wong et al. [28]. The cooked corn was steeped for 14 h, then the cooking liquor (nejayote) was drained and the cooked kernels (nixtamal) were washed with tap water to remove the excess calcium and dissolved solids.

#### 2.3.2. Grinding and Mixing

The nixtamal was ground to obtain fresh corn dough (fresh masa) in a 1HP volcanic stone spiral mill with a 5” diameter (Maquinaria del Río SA de CV, Michoacán, Mexico). Water was added subjectively to the fresh masa and mixed for 3 min to obtain a suitable masa consistency. Part of the fresh masa was lyophilized and ground to obtain nixtamalized corn flour (NCF). The other part of the masa (fresh masa) was used to make tortillas.

#### 2.3.3. Tortilla Preparation

Corn tortillas from the TNP were made with fresh masa in the same commercial plant as described with same processing conditions previously described for the ENP. Then, the tortillas were cooled and transported to the laboratory and stored at room temperature (25°C) for further analysis.

### 2.4. Analytical Evaluations

Measurement of the moisture content, pH, and resistant starch (RS) content of corn flour, masa, and tortilla from both processes was performed following the AACC [24] International Approved Methods 44–19.01, 02–52.01, 32–40.0. The RS content (Megazyme, K-RSTAR 08/15) was reported as g RS/100 g. All determinations were made in triplicate.

#### 2.4.1. Scanning Electron Microscopy (SEM)

The morphology of the different samples was evaluated according to the procedure reported by Sánchez-Madrigal et al. [10], as follows: samples of ground corn grain, flour, masa, and tortilla from both nixtamalization processes were imaged with a scanning electron microscope (JSM-5800LV, JEOL, Akishima, Japan) and were exposed to an acceleration rate of 10 kV. Samples with particle sizes <0.15 mm and a moisture content of 1% were fixed and coated with a gold layer in a vacuum evaporator (Denton Desk II) at a pressure of 7.03 × 10^–2^ kg/cm^2^ using 1000X magnification.

### 2.5. Corn Flour Evaluations

#### 2.5.1. Particle Size Distribution (PSD)

Determination of the PSD of fresh masa from the TNP and the extruded nixtamalized corn flour of the ENP was carried out on a set of sieves but on a wet basis. This obeys to that one flour (ENCF) sample and the other semisolid (fresh masa). In this case the masa fractionation technique reported by Pflugfelder et al. [29], was used. Briefly, for the TNP, 50 g of fresh masa were gently slurried with 100 mL of distilled water in a flask. The slurry was resuspended and washed with water through U.S. standard sieves No. 20 (850 *µ*m), 30 (600 *µ*m), 40 (425 *µ*m), 60 (250 *µ*m), 80 (180 *µ*m) and bottom. Separation of fractions was performed in a vibratory shaker (AS 200, Retsch GmbH, Haan, Mettmann; Germany) for 5 min. Each sieve fraction was placed in a tared weighing aluminum dish and dried in an oven at 110°C for 24 h. The percentage of material retained in each sieve was calculated.

Regarding the ENP, water was added to a sample of extruded nixtamalized corn flour to make masa with a moisture content to prepare tortillas (53.1%). Then 50 g of masa were taken and the masa fractionation was done in the same way as the procedure to fractionate fresh masa of the TNP.

#### 2.5.2. Particle Size Index (PSI)

The PSI calculation followed the methodology reported by Bedolla and Rooney [30] and was performed in triplicate using the following formula:

(1) $$\begin{array}{c} \text{PSI}=\sum \left(\left(\#{\text{SF}}_{i}\right)\left(\%{\text{RM}}_{i}\right)+\cdots +\left(\#{\text{SF}}_{n}\right)\left(\%{\text{RM}}_{n}\right)\right), \end{array}$$

where #SF is the sieve factor number and %RM_*n*_ is the retained material in each sieve.

The factor number depends on the U.S. sieve number (0.2, sieve n° 20; 0.3, sieve n° 30; 0.4, sieve n° 40; 0.6, sieve n° 60; 0.8, sieve n° 80; and 1.0, bottom).

#### 2.5.3. Water Absorption Capacity (WAC)

The WAC calculated was according to the criteria in Flores-Farias et al. [31], and reported as mL of water/100 g flour and in triplicate.

#### 2.5.4. Water Absorption Index (WAI)

The WAI was calculated from both corn flours according to the procedure of Anderson et al. [32] with slight modifications as follows: a 1 g sample (previously lyophilized and ground) was mixed with 15 mL of distilled water in a 50 mL centrifuge tube at 25°C. The suspension was stirred for 30 min and centrifuged at 5000 rpm for 30 min. The supernatant was placed in a tared aluminum plate and evaporated in a convection oven at 105°C for 12 h. The weight of the gel, as well as the precipitate, was registered, and the WAI was reported as g gel/g dry sample and in triplicate.

#### 2.5.5. Water Solubility Index (WSI)

The WSI was determined using the methodology reported by Anderson et al. [32], in triplicate using the formula:

(2) $$\begin{array}{c} \text{WSI}=\left(\frac{\text{WSMS}}{\text{ISW}}\right)*100, \end{array}$$

WSMS is the weight of soluble material in the supernatant and ISW is the initial sample weight.

### 2.6. Masa Rheological Evaluations

#### 2.6.1. Frequency Sweep Test

The dynamic frequency sweep test was performed to measure the viscoelastic moduli of masa from both nixtamalization processes (Rheometrics Model RSF III, Piscataway, NJ, USA). The elastic (*G*′) and viscous (*G*^″^) moduli of masa from both processes were obtained according to the procedure reported by Platt-Lucero et al. [13]. Briefly, a sample (3.0 g) was placed between two parallel plates with a diameter of 25 mm and a 2.5 mm gap. Part of the masa that was exposed to the environment was covered with petroleum jelly to avoid loss of moisture. The frequency sweep test was executed using a strain of 0.04% (linear viscoelastic region) at 25°C and a frequency range between 0.1 and 100 rad/s.

#### 2.6.2. Temperature Sweep Test

The dynamic temperature sweep test was performed to evaluate the changes in the viscoelastic properties of the mass of both nixtamalization processes (Rheometrics Model RSF III, Piscataway, NJ, USA). The elastic modulus (*G*′) and the viscous modulus (*G*^″^) of masa from both processes were obtained according to the procedure reported by Gerardo-Rodríguez et al. [33]. Briefly, a sample (3.0 g) was placed between two parallel plates with a diameter of 25 mm and a 2.5 mm gap. Part of the masa was exposed to the environment, after which Silicon oil (Sigma Aldrich, U.K.) was placed on the edges of the masa to help avoid moisture loss. The temperature sweep test was executed in the temperature range from 25°C to 120°C with a frequency of 5 rad/s and 0.04% strain (linear viscoelastic region). The viscoelastic parameters determined the *G*′ and *G*^″^ moduli.

### 2.7. Tortilla Evaluations

#### 2.7.1. Physical Properties

Ten tortillas from each process were sampled to evaluate their weight (g), diameter (cm), and thickness (mm). Tortilla weight measurements were performed on an analytical balance (Sartorius Research R300S), while diameter and thickness were evaluated with a digital Vernier (Model CD6”C Mitutoyo Corporation, Kanawa, Japan). Mean values and the standard deviation of each physical property were reported.

#### 2.7.2. Firmness

To evaluate the firmness and rollability at 2, 24, and 48 h of storage at room temperature, a procedure suggested by the corn flour industry was used, which indicates that the texture of a tortilla should be evaluated as a hot tortilla instead of room temperature (personal communication). Tortillas were preheated as follows: individual pieces of tortilla were placed in a polyethylene bag and then heated in a microwave oven (Samsung Co., México) for 15 s at 100% electrical power. Immediately after heating, the tortilla was cut into a 41.47 cm^2^ rectangle and placed on the lower plate of the Kramer cell (part code HDP/K55 Stable Micro Systems, Surrey, England). Once the temperature of the tortilla reached 30°C, the piece of tortilla was sliced with a five-bladed Kramer cell (top part) and then was connected to a texture analyzer (TA-XT-Plus Stable Micro Systems, Surrey, England). The head speed was set at 2 mm/s and the firmness was reported in kPa [34].

#### 2.7.3. Rollability

The subjective method reported by Arámbula-Villa et al. [14], was used to assign a nominal score for the measurement of tortilla rollability. A score of 5 indicates no tortilla breakage (the best rollability), a score of 3 reflects 50% breakage in tortilla structure, and a score of 1 represents 100% tortilla breakage. The tortilla preparation was identical to the procedure reported to evaluate tortilla firmness. The rollability was measured at 2, 24, and 48 h of storage.

### 2.8. Experimental Design and Statistical Analysis

A completely randomized experiment was used, where the factor was the type of nixtamalization process (TNP or ENP) applied to each product (corn flour, masa and tortilla). Analysis of variance (ANOVA) was performed on all data gathered from the different evaluations using a significance level of 95%. To evaluate significant differences among the specific means, Tukey's test was performed with 95% significance. ANOVA was performed using the Statistical Analysis Software [35].

## 3. Results and Discussion

### 3.1. Physicochemical Properties of Corn Flours

Table 1 shows the physicochemical properties of the flours for the extrusion nixtamalization process and traditional nixtamalization processes. Both procedures gave products with different characteristics. TNP produced masa with a moisture content of 53.1% (g/100 g flour) and ENP produced corn flour with a moisture content of 8.8% (g/100 g flour). To perform the same evaluations, the moisture content of masa was freeze-dried and diminished to a final value of 3.4%. According to this behavior, the ANOVA showed very significant differences (*p* < 0.01) between the moisture contents of both flour types.

Regarding the pH, corn flours from both processes had an alkaline pH, which was slightly but significantly (*p* < 0.05) higher in TNP (Table 1). This pH is desirable in tortillas because of the traditional alkaline flavor identified by consumers [29].

Resistant starch****content values were higher (*p* < 0.05) for extruded flour (Table 1) than for freeze-dried nixtamalized flour. This is probably due to the higher amount of fiber contained in whole corn meal coming from dry milling. The reduced water content, temperature inside the extruder, and subsequent periods of heating and cooling prevent complete gelatinization. Thus, macromolecules such as amylopectin are partially disrupted and short lineal chains appear, promoting a low enzymatic digestion of starch [21, 36, 37].

Although a stronger interaction between starch and calcium is observed in the thermoalkaline process, the RS content can decrease because of pericarp losses in nejayote. Amylose leached, and gradual gelatinization gives rise to a stable crystalline structure, which takes more time. Thus, the gelatinization temperature and enthalpy are bigger, and a higher crystallinity was observed [38]. The RS values for both flours in this study are in agreement with Villada et al. [39], and Gutiérrez-Dorado et al. [21].

#### 3.1.1. Particle Size Distribution

**Figure 1:**
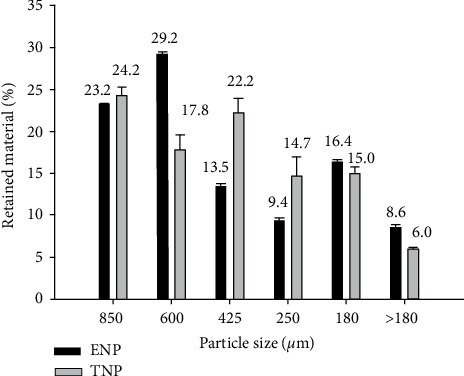
Particle size distribution of masa from the ENP and TNP. Bars indicate standard deviation.

Figure 1 presents the fraction of material in percentage of masa from the TNP and ENP retained in each mesh. According to the ANOVA results, significant (*p* < 0.05) differences between nixtamalization processes were observed in every fraction of retained material. In both types of masa, the retained accumulative material between sieve U.S. No. 20 (850 *μ*m) and sieve U. S. No. 80 (180 *μ*m) were 92.3% and 83.1% for ENP and TNP, respectively. In this way, Mexican regulations related to the production of nixtamalized corn flours were accomplished when up to sieve U.S. No, 80 (180 *μ*m), 75% of the accumulative material is retained [40]. This indicate that this PSD has coarse to medium distribution of particles, which is appropriate for making tortillas. The masa fractionation method allowed an efficient comparison between both processes.

The PSD is directly related to the milling process used. Dry milling produces fine granulometry, modifying the physicochemical characteristics and drastically changing the granular structure and surface area of starch [5]. Wet milling requires coarse and fine particles to produce corn flour, but these differences are less pronounced than dry milling.

According to Campas-Baypoli et al. [41], Gómez and Vaniska [42], and Gómez et al. [43], it is possible to measure the extent of gelatinization by monitoring the presence of native and fragmented starch granules in every step of both processes. SEM micrographs show the quantity of damaged starch granules in corn grain, flour, masa, and tortilla. As operations progress in both nixtamalization processes, the damage to starch increases, being more critical during baking of masa to tortilla. This can be shown in a scanning electronic micrograph.

Table 1 shows the particle size index (PSI) of the corn flours from the ENP and TNP. According to Bedolla and Rooney [30], the higher the PSI value, the finer the corn flour. The PSI in ENCF was significantly (*p* < 0.05) higher than NCF. This is probably due to the milling process used. Dry milling is more abrasive and produces a finer particle distribution, therefore creating more damaged starch granules. In contrast, wet milling is responsible for creating coarse granulometry, where more native and fragmented starch granules are present in every stage of the process [44]. Therefore, the cohesiveness of the masa is increased and the machinability of the tortillas improved. Choosing an adequate PSI to produce flour allows the improvement of the extrusion process conditions.

#### 3.1.2. Water Absorption Capacity

The WAC (L water/kg flour) subjectively measures the amount of water absorbed by the flour during masa preparation; this is the quantity of starch degraded in the extrusion process [31]. Extruded nixtamalized corn flour exhibited a lower WAC (*p* < 0.05) than did nixtamalized corn flour (Table 1). This is probably due to the higher cooking degree and the compaction of extruded flour as a consequence of the smaller intermolecular spaces.

Dry milling and thermal gelatinization conditions are more severe than those observed in wet milling. Thermomechanical processes force the gelatinization in an accelerated manner, lowering the water absorption capacity of the extruded flours. Contreras-Jimenez et al. [22], considered the particle size, gelatinization degree, and damaged starch content as the most important factors for increasing the WAC in extruded flours.

However, wet milling is performed using plenty of water, which protects the starch granule structure. The corn grain steeping period when cooking liquor promotes gradual starch granule gelatinization and higher calcium absorption, hardening the cell wall. For that reason, the water enters into the protein matrix more easily.

We have found it necessary to develop extrusion conditions that do not damage the starch as extensively to increase the water absorption capacity; consequently, the textural and sensory characteristics of masa and tortillas are more like those from the traditional process.

#### 3.1.3. Water Absorption Index

The WAI value relates with milling and extrusion parameters such as barrel temperature and moisture content. Extruded nixtamalized corn flour presented a slightly but higher water absorption index (*p* < 0.05) than nixtamalized corn flour (Table 1). This is probably due to the limited water content used in dry milling, producing a higher fragmentation of starch granule and dextrinization [13, 20]. In such a way, the breaking of inter and intramolecular hydrogen bonds allows the release of hydroxyl groups and increase the capability to form more hydrogen bonds with water [20].

Comparing both dry milling and wet milling, a high WAI is required to promote more flexibility and a better reheating capability in tortillas [45] but, excessive heating contribute to form an amorphous and sticky masa; where starch granules lose structure and integrity [19]. Therefore, appropriate processing conditions in both processes help to diminish the damaged starch content. Similar WAI values have been reported previously [1, 4, 6, 13].

#### 3.1.4. Water Solubility Index

The WAI value is related to milling and extrusion parameters such as the barrel temperature and moisture content. Extruded nixtamalized corn flour presented a slightly higher water absorption index (*p* < 0.05) than nixtamalized corn flour (Table 1). This is probably due to the limited water contents used in dry milling, which produces higher starch granule fragmentation and dextrinization [22, 46]. In this way, the breaking of inter and intramolecular hydrogen bonds allows the release of hydroxyl groups and increases the ability to form more hydrogen bonds with water [20].

By comparing dry milling and wet milling, a high WAI is required to promote more flexibility and better reheating of tortillas [1], but excessive heating contributes to the formation of amorphous and sticky masa, in which starch granules lose their structure and integrity [41]. Therefore, appropriate processing conditions in both processes help to diminish the damaged starch contents. Similar WAI values have been reported previously [6, 20, 21, 39].

### 3.2. Masa Physicochemical Evaluations

#### 3.2.1. Moisture Content, Resistant Starch, and pH

The moisture contents of masa were significantly higher (*p* < 0.05) than masa prepared with extruded flour (Table 2).

This occur because extruded flour absorbed less water than masa. The extreme conditions inside the extruder are probably the main causes why the starch granules do not increase in volume as much as during traditional nixtamalization. In dry milling, the thermal and mechanical damage is higher. Starch granules do not adequately expand, producing a higher flour density, which leads to a more compact product.

Wet milling eases the release of starch granules from the protein matrix [36]. This allows the increase in the water content because of the starch granule swelling, resulting in an increase in the volume up to 30−40%. Another point to consider is that annealing favors the interaction of starch granules with calcium during the steeping of cooked corn, which is between 12 and 14 h. Annealing is performed in a range between the glass transition temperature and the gelatinization temperature. This mechanism promotes an increase in the water content in masa to approximately 45−60% (w/w) and improves the pasting, rheological, and textural properties of masa [47].

Table 2 shows the resistant of starch contents from both the nixtamalization processes. RS was higher in the extruded nixtamalized corn flour, though no significant differences was observed. This increase is probably due to the grinding method used to obtain the corn flour. Dry milling of whole corn grain includes the pericarp and avoids the release of lipid and proteins from starch granules; therefore, it increases the dietary fiber content. Gelatinization still occurs though there is a reduced amount of water present.

Wet milling refers to the transformation of nixtamalized corn grain into fresh masa considering the loss of fiber and lipids during nejayote drainage. This reduces the formation of amylose-lipid complexes. Villada et al. [39], found similar RS contents in extruded flour, which is consistent with the behavior observed in this study.

Although the measurement of pH in both processes were significantly different (*p* < 0.05), both the alkaline values mean that specific lime content is necessary to promote the correct flavor when the tortilla is produced.

### 3.3. Masa Rheological Evaluations

#### 3.3.1. Frequency Sweep Test

**Figure 2:**
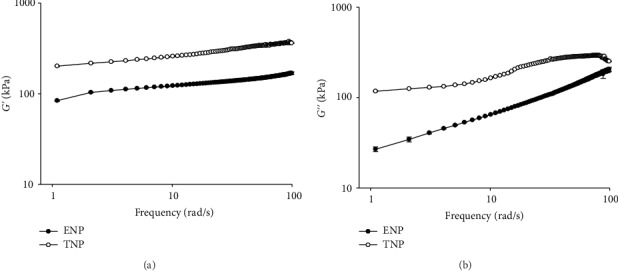
(a) Elastic modulus (*G*′) and (b) viscous modulus (*G*′) of corn masa as a function of the frequency from the extruded (ENP) and traditional (TNP) nixtamalization processes.

Figure 2 shows the viscoelastic parameters *G*′ and *G*^″^ as a function of frequency. According to Figure 2(a), *G*′ showed a linear increase up to 10 rad/s. Then, the frequency rate increased and the structure of polymers collapsed [48]. The higher *G*′ observed in fresh masa is probably due to the lime concentration used during traditional nixtamalization. In contrast, wet milling includes the treatment of the starch granule with a higher concentration of lime, promoting stronger interactions with calcium ions, and leading to gradual gelatinization [33]. Moreover, the steeping period in cooking liquor reduces the glass transition temperature and imparts mobility to the starch granule amorphous region, making them softer, deformable, and possibly more elastic [48]. However, the dry milling and thermomechanical process of extrusion destroys the starch granule native structure, which occurs with little water content and hastens gelatinization. This leads to the decrease in the *G*′ value [33].

Regarding the behavior of *G*^″^ (Figure 2(b)), the lower values observed in dough are the result of a higher quantity of pregelatinized starch. When extruded dough is rehydrated, it presents a compact and solid-state that originates due to the small difference between the viscoelastic moduli, which is a characteristic of a Hook solid. Dry milling and the thermomechanical processes produce a dough with less resistance and lower viscoelastic properties.

Another apparent reason explaining this behavior is that dry milling induces deeper disruption of the crystalline structure and starch molecules degradation [12].

It can be assumed that the higher water content of the nixtamalized corn flour increases the elastic behavior of masa. Wet milling implies the formation of an elastic network due to a crystalline domain derived from the complexation reaction between amylose and lipids. Therefore, the viscoelastic moduli show a large difference and the elasticity is increased.

#### 3.3.2. Temperature Sweep Test

**Figure 3:**
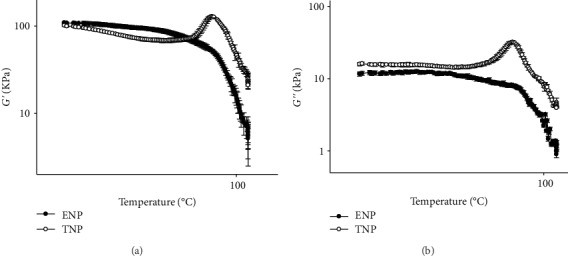
(a) Elastic modulus (*G*′) and (b) viscous moduli (*G*^″^) of corn masa as a function of temperature from the extruded (ENP) and the traditional (TNP) nixtamalization processes.

Figure 3 shows the viscoelastic behavior of masa from the ENP and TNP as a function of temperature. Fresh masa showed a higher *G*′ than the extruded dough, indicating the formation of two different starch granule structures (Figure 3(a)). The maximum peak detected (129 KPa) in the *G*′ plot of fresh masa is between 76°C and 80°C which explains the starch gelatinization of native and fragmented starch granules. This is probably due to the jamming of small-sized particles obtained in the fine grounding of the corn flour. This also explains how some granules remain undamaged during this step in the process [34].

The extruded masa did not present any peaks, and as temperature increased, the *G*′ curve showed a downward trend (Figure 3(a)). A drop in the *G*′ indicated a higher weakening in the network structure. The straight line observed in the extruded dough plot probably indicates the presence of pregelatinized starch and the decrease in the highly branched structure of amylopectin chains due to shear and heat degradation [33].

The results for the elastic moduli were similar to those obtained for the viscous moduli (*G*^″^) for both processes (Figure 3(b)). The *G*^″^ of fresh masa displayed a gelatinization temperature range between 76°C and 80°C, a maximum peak value of 32 kPa, and an abrupt decay when gelatinization concluded. In contrast, *G*^″^ of extruded masa decreased with the increase in temperature rise and did not show any peak.

This pronounced reduction in the viscous modulus of extruded masa is related to the rapid gelatinization and the previous modification of the starch granule structure [12]. *G*′ was greater than *G*^″^ for both processes, indicating the predominance of the elastic contribution over the viscous contribution.

We have found the temperature sweep test as a very useful tool because of rheological changes, mainly the peaks observed for the elastic and viscous moduli, are clearly attributable to changes in starch granules due to gelatinization, and allow us to graphically observe the differences in the degree to which starch changes are produced by both processes. Manipulating the WAC and WAI is a way to control extrusion conditions so that physicochemical starch changes would be similar in both processes. When we observe similar behaviors in the sweep test for the masa produced from extruded nixtamalized corn flour and fresh masa produced by the traditional nixtamalization process, we will identify extrusion conditions where the starch changes are similar, which will give rise to tortillas with better textural and organoleptic properties that are obtained through the extrusion process.

### 3.4. Tortilla Physicochemical Properties

#### 3.4.1. Moisture Content

The moisture content in the tortillas from the ENP was significantly (*p* < 0.05) higher than from the TNP (Table 3). This difference is probably due to the amount of water added by the operator. When dough and masa were prepared previously in the laboratory, an empirical moisture content was established. From this moment on, the measurement of subjective texture was the responsibility of the operator. Tortillas from both processes were prepared at the same commercial plant with the same baking process conditions. Proper temperature, water content, cooking time, and drying allowed for enhanced masa and tortilla textural properties.

The moisture content of the corn tortilla is related to the water absorption capacity (WAC) and water absorption index (WAI), which is dependent on the damage to the starch granules [18].

#### 3.4.2. Resistant Starch

Table 3 shows RS contents in tortillas made with extruded nixtamalized corn flour and fresh masa. This value was significantly higher (*p* < 0.05) in tortillas made with ENCF (1.65 g/100 g sample) than with fresh masa (0.90 g/100 g sample). This behavior is probably due to the processing steps in the extrusion nixtamalization process, such as dry milling, cooking, and baking, where starch granules are exposed to more severe mechanical and thermal treatment than that used to obtain an extruded corn meal [49]. The high RS in tortillas produced with ENCF is also related to the higher dietary fiber content in the pericarp [21].

In contrast, the production of RS in the traditional nixtamalization process occurs during the cooking, steeping, masa formation, and tortilla baking steps. The RS content may decrease or increase according to the degree of starch gelatinization and type of resistant starch [47]. Similar RS values for corn flours reported previously [21, 41] agree with this study.

Retrogradation is directly related to the RS content. The reorganization reactions between amylose and amylopectin in retrograded starch depend on the storage time and ability of these polymers to form a new crystalline network. Amylose affects the short-term storage and is responsible for hardening and the loss of flexibility in tortillas. Amylopectin has long-term effects and is related to the recrystallization process [41].

#### 3.4.3. Physical Properties

Table 3 shows the weight, diameter, and thickness of tortillas made with both technologies. Tortillas made with ENCF had significantly (*p* < 0.05) higher weight, diameter, and thickness values than those observed for fresh masa. This is probably due to the whole corn milled to produce the ENCF. Therefore, the diameter and thickness of the dough disks can be increased. According to Mery et al. [50], the corn grit particle size and masa disk thickness are the most important attributes in the subjective measurement of tortilla quality.

#### 3.4.4. Firmness

**Figure 4:**
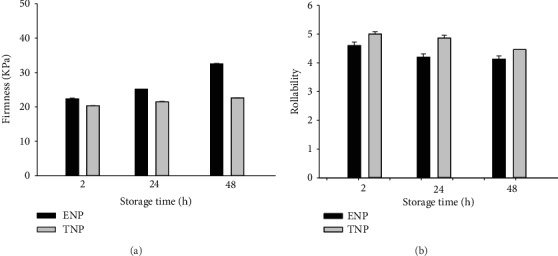
(a) Firmness and (b) rollability of corn tortillas from the extruded (ENP) and traditional (TNP) nixtamalization processes during storage. Bars indicate standard deviation.

Figure 4(a) presents the firmness of tortilla evaluated at 2, 24, and 48 h of storage time at room temperature (25°C). Tortillas were preheated (60°C) and then cooled to 30°C before the firmness measurement. This heating process suggested by the corn flour industry (personal communication) allows the measurement of firmness in the usual way a tortilla is consumed. The ANOVA indicated that tortillas made with ENCF were significantly harder (*p* < 0.05) than those made with fresh masa. Firmness increased to 18% and 50% in extruded tortillas and 0.75% and 1.25% in nixtamalized tortillas after 24 and 48 h of storage, respectively. This is probably due to the processing conditions used during extrusion and the changes in the gelatinization, melting, and dextrinization that directly affect the texture of the starch gels [20]. Another explanation is the effect of the milled whole corn fiber content used in the ENP.

Alam et al. [51], mentioned that flours with a smaller particle size result in a starch-fiber matrix with a rigid structure, and therefore a harder product. Hardness is likely the sensory attribute that is most affected by extrusion and associated with the texture of tortillas. Firmness values obtained for ENCF reported by Platt-Lucero et al. [13], and Gutiérrez-Dorado et al. [21], behaved in a similar manner.

#### 3.4.5. Rollability

Rollability is a common subjective test related to the starch gelatinization reached during nixtamalization, and it is affected by the lime concentration and cooking time [44]. Rollability values are measured by assigning a score that reflects the tortilla breakage and somehow measures the starch damage.

Figure 4(b) shows the tortilla rollability at 2, 24, and 48 h of storage at room temperature (25°C). The ANOVA showed significant differences (*p* < 0.05) at 2 h and very significant differences (*p* < 0.01) after 24 h between both types of tortilla. The measurement at 48 h did not show significant differences (*p* > 0.05). This is probably due to the retrogradation process undergone by all types of tortillas. For both processes, tortillas were more breakable as the storage time progressed. The ANOVA also showed that the type of process and the storage time very significantly affected the rollability (*p* < 0.01). The interaction between both variables (process *×* time) was not significant (*p* > 0.05).

The tortilla reheating process was similar to that reported in the tortilla firmness section. This procedure allowed us to obtain high rollability values, leading to a score above 4, and in our opinion, both tortilla types were adequate for the consumer. Other studies showed that rollability of tortillas made with ENCF without a previous reheating process diminished drastically as the storage time increased [11, 13, 14].

### 3.5. Morphology

**Figure 5:**
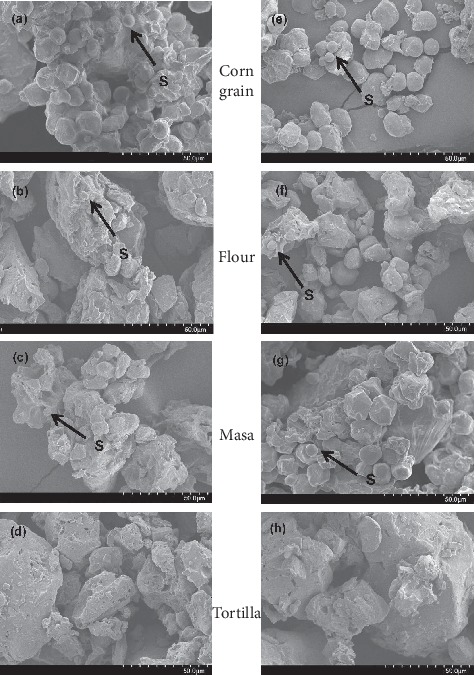
(a, e) SEM images of corn, (b, f) flour, (c, g) masa, and (d, h) tortilla from ENP and TNP. Magnification is 1000X.

Figure 5 shows the scanning electron micrographs (SEM) images of corn grain, flour, masa, and tortilla from the ENP (Figures 5(a), 5(b), 5(c), and 5(d), respectively) and TNP (Figures 5(e), 5(f), 5(g), and 5(h), respectively). The presence of native and fragmented starch granules in each step of both processes was observe. Figures 5(a) and 5(e) show SEM images of corn, the starch granules (*S*) are round and polyhedral in shape with smooth surfaces and minimal depressions and a wide distribution before processing. Starch granules with a smooth surface or with some depressions were also observed [41].

Figures 5(b) and 5(f) show SEM images of extruded nixtamalized corn flour from ENP and freeze-dried masa from TNP, respectively. With respect to the ENCF, Figure 5(b) shows the reduction in the number of granules and the increase in the irregular shape and pore surface for both treatment, but was higher in the extruded flour corn, probably due to higher enzymatic hydrolysis and starch digestibility [52]. Dry milling of corn kernels occurring in extrusion-cooking is carried out with low moisture contents and causes a high amount of starch granules to be fragmented and embedded in the endosperm matrix, as well as that some granules to be dispersed out of it. This agglomeration of SD granules results in amorphous structures, and at this point, dextrinization is possible [10, 51].

On the other hand, Figure 5(f), shows the SEM images of freeze-dried flour from TNP, like ENP, a reduction in the number of granules and irregular shapes surfaces. However, its general effect on the flour is less in TNP than ENP. The reduction in particle size for both flours increases the porosity and reduces the cellular connectivity, which directly affects the dough (masa) and the tortilla textural parameters [47]. Sánchez-Madrigal et al. [10], observed an extensive area of damaged starch granules in corn flour during the extrusion process.

Figures 5(c) and 5(g) show SEM images of masa from the ENP and TNP, respectively. Masa obtained with ENCF in Figure 5(c) presents almost null presence of native starch granules with a defined structure. The extrusion-cooking process originate more with SD and an amorphous structure due to the high extent of hydrolysis of the gelatinized starch [53]. In this step, it is important to control the mixing time and the addition of water to avoid adhesive extruded dough [12]. In contrast, Figure 5(g) shows nixtamalized masa with fragmented starch granules and some without fragmentation in the native state. The enzymatic attack is prevented by wet milling [25]. The structures obtained are more defined due to the partial gelatinization that the granule undergoes during annealing and retrogradation [39]. The formation of resistant starch is increased on through the nixtamalization process and allows some granules to remain with its native crystalline structure, and others completely collapsed [30].

Figures 5(d) and 5(h) show SEM images of tortillas from the ENP and TNP, respectively. For both extrusion and traditional nixtamalization, the production of tortillas presents continuous amorphous structures in the form of agglomerates [25]. This is a consequence of the fusion of starch granules during the thermal processing due to high temperatures (270–290°C). In this stage, the complete gelatinization of starch granules occurs, the volume of starch granules decreases considerably, and only a small number of starch granules are present [36, 54]. Finally, it can be seen that the greatest damage to the starch granules for the ENP is during extrusion to produce ENCF and in masa-tortilla baking step. In TNP, the greatest damage of starch is in masa-tortilla baking step.

## 4. Conclusion

In general, the physicochemical, rheological, and morphological characteristics of products from the traditional and extrusion nixtamalization processes were different from the nixtamalization extrusion process. This may be the reason that there is more damage in corn starch in EPN than TNP. Corn flour from the TNP had lower PSI, and resistant starch but higher water absorption capacity than flour from the ENP. The viscoelastic parameters *G*′ and *G*^″^ as a function of frequency were higher in masa from the TNP than masa from the ENP. According to results of the temperature sweep test, Fresh masa from TNP showed a peak in *G*′ (364 KPa) and in *G*^″^ (138 KPa), while masa from ENP had lower values of these peaks (*G*′ = 113 KPa, *G*^″^ = 33.5 KPa). Tortillas from the traditional nixtamalization process showed lower hardness and higher rollability values after 24 h of storage. At 48 h of storage, similar values of those parameters were found in both processes. Extrusion proved to be a good alternative in the production of corn flour and tortillas, although dry milling produced (ENP) more starch damage than wet milling (TNP). The resistant starch content in corn flour, masa, and tortillas increased gradually in both processes, but were higher in ENP than TNP. This represents nutrimental benefits in health for tortilla consumers. Finally, the greatest starch damage in the ENP is during the extrusion step to produce flour and in masa-tortilla baking step. In TNP, the greatest damage of starch is in masa-tortilla baking step.

## Figures and Tables

**Table 1:** Physicochemical characteristics of corn flour from the extruded (ENP) and traditional (TNP) nixtamalization processes.

Characteristic	ENP	TNP
pH	8.2^1^ ± 0.11a^2^	8.4 ± 0.06b
Moisture content (%)	8.83 ± 0.39a	3.42 ± 0.06b
Particle size index	51.01 ± 0.57a	48.7 ± 0.32b
WAC^3^ (mL water/100 g flour)	104.2 ± 0.29a	108 ± 0.5b
WAI^4^ (g gel/g dry matter)	3.7 ± 0.08a	3.6 ± 0.06b
WSI^5^ (%)	5.8 ± 0.01a	3.6 ± 0.06b
Resistant starch (g/100 g sample)	1.01 ± 0.051a	0.79 ± 0.06b

^1^Means ± standard deviation. ^2^Means with the same letter are not significantly different (*p* > 0.05). ^3^WAC Water absorption capacity. ^4^WAI Water absorption index. ^5^WSI water solubility index.

**Table 2:** Physicochemical characteristics of corn masa from the extruded (ENP) and traditional (TNP) nixtamalization processes.

Characteristic	ENP	TNP
Moisture content (%)	53.1 ± 0.13a^1,2^	57.4 ± 0.68b
Resistant starch (g/100 g sample)	1.43 ± 0.053a	0.85 ± 0.073b
pH	8.2a	8.4b

^1^ Means ± standard deviation. ^2^ Means with the same letter are not significantly different (*P* < 0.05).

**Table 3:** Physicochemical and physical characteristics of corn tortillas from the extruded (ENP) and traditional (TNP) nixtamalization processes.

Characteristic	ENP	TNP
Moisture content (%)	45.0 ± 0.18a^1^	40.7 ± 1.63b
Resistant starch (g/100 g sample)	1.64 ± 0.06a	0.90 ± 0.04b
*Physical characteristics*		
Weight (g)	26.81 ± 1.0a^2^	20.3 ± 1.57b
Diameter (cm)	14.0 ± 0.14a	13.9 ± 0.09b
Thickness (mm)	2.5 ± 0.1a	1.4 ± 0.11b

^1^Mean ± standard deviation. ^2^ Mean with the same letter are not significantly different (*p* > 0.05).

## Data Availability

All the experimental data used to support the findings of this study are available from the corresponding author upon request.
